# Clinical outcomes of kinematically aligned medial pivot total knee arthroplasty: A systematic review and meta‐analysis of current evidence

**DOI:** 10.1002/jeo2.70348

**Published:** 2025-07-13

**Authors:** Francesco Bosco, Giorgio Cacciola, Virginia Masoni, Carmelo Burgio, Mariazzurra Carlino, Michele Centola, Ferdinando Tosto, Daniele Vezza, Lawrence Camarda, Luigi Sabatini

**Affiliations:** ^1^ Department of Precision Medicine in Medical, Surgical and Critical Care (Me.Pre.C.C.) University of Palermo Palermo Italy; ^2^ Department of Orthopaedics and Traumatology G.F. Ingrassia Hospital Unit Palermo Italy; ^3^ Department of Orthopaedics and Traumatology, University of Turin CTO Turin Italy; ^4^ Adult Reconstruction and Joint Replacement Service Hospital for Special Surgery New York New York USA; ^5^ Department Statistics, Economics and Business,Alma Mater Studiorum University of Bologna Bologna Italy; ^6^ Department of Orthopaedics, Gradenigo Hospital Humanitas Turin Italy

**Keywords:** biomechanics, kinematic alignment, medial pivot, posterior cruciate ligament, total knee arthroplasty

## Abstract

**Purpose:**

Kinematic alignment (KA) in total knee arthroplasty (TKA) focuses on restoring the natural joint line and ligament balance, potentially improving biomechanics and outcomes over mechanical alignment (MA). The medial pivot (MP) implant enhances joint stability by mimicking physiological knee motion. Still, its role within a KA protocol and the effects of retaining versus sacrificing the posterior cruciate ligament (PCL) are unclear. This study aimed to evaluate the clinical effectiveness, functional outcomes and biomechanical benefits of KA‐TKA with MP implants based on the available literature. It also aimed to assess whether PCL retention or sacrifice leads to better postoperative function.

**Methods:**

A systematic review and meta‐analysis were conducted following PRISMA guidelines. A comprehensive search of PubMed, Embase and Web of Science up to January 2025 identified studies assessing KA‐TKA with MP implants. Primary outcomes included patient‐reported outcome measures (PROMs), range of motion (ROM), complication rates and implant survivorship. Subgroup analysis compared PCL retention and sacrifice.

**Results:**

Fourteen studies (955 patients) met inclusion criteria. KA‐TKA with MP implants resulted in significant ROM improvements (+11.35°, increased to +12.50° after sensitivity analysis) and enhanced PROMs (Oxford Knee Score +18.23, increased to +22.27 after sensitivity analysis; Knee Society Score [KSS] +49.61, functional KSS +42.50). No aseptic loosening or implant failures were reported. PCL sacrifice was associated with greater postoperative flexion (125.4° ± 12.1° vs. 116.4° ± 11.8°, *p* < 0.001), but functional outcomes were comparable.

**Conclusions:**

KA‐TKA with MP implants improves functional recovery, patient satisfaction, and short‐ to mid‐term survivorship, supporting its adoption as a viable alternative to conventional TKA. Further long‐term, randomized trials are needed to optimize PCL management and confirm its durability.

**Level of Evidence:**

Level IV.

**Abbreviations**
BMI: body mass index
FJS: Forgotten Joint Score
fKSS: functional Knee Society Score
KA: kinematic alignment
KSS: Knee Society Score
MA: mechanical alignment
MP: medial pivot
OA: osteoarthritis
OKS: Oxford Knee Score
PCL: posterior cruciate ligament
PRISMA: Preferred Reporting Items for Systematic Reviews and Meta‐Analyses
PROMs: patient‐reported outcome measures
ROBINS‐I: Risk of Bias In Non‐randomized Studies of Interventions
ROM: range of motion
SD: standard deviation
TKA: total knee arthroplasty

## INTRODUCTION

Knee osteoarthritis (OA) is one of the leading causes of disability worldwide, significantly impacting the quality of life of affected patients [13, 30]. Total knee arthroplasty (TKA) is the definitive surgical treatment for advanced OA, aimed at reducing pain and restoring joint function [13, 30]. However, 20%–25% of patients report postoperative dissatisfaction, often attributed to the failure to replicate the knee's natural kinematics [13, 30].

Mechanical alignment (MA) has long been considered the standard reference in TKA surgery to ensure uniform load distribution and biomechanical stability over time [13, 28, 30, 42]. However, recent studies have questioned its ability to restore physiological joint movement, highlighting how MA may alter joint dynamics and compromise patient comfort [13, 21, 28, 30, 42]. In this context, kinematic alignment (KA) has emerged as a promising alternative to preserve pre‐arthritic anatomy and native joint kinematics [3, 21, 23, 28, 30].

Recent literature has demonstrated that KA can offer advantages in terms of joint functionality, range of motion (ROM) and the perception of a more natural knee compared to MA [10, 34]. Meta‐analyses and randomized control trials suggest that KA reduces the need for ligament releases, improves functional outcomes, and does not increase the complication rate compared to MA [13, 30, 32, 51]. However, uncertainties remain regarding its long‐term durability, particularly in patients with severe deformities [23, 28, 30, 42].

Specific prosthetic designs, such as medial pivot (MP) implants, further enhance the benefits of KA by optimizing stability and load distribution [3, 26]. Three‐dimensional registration analysis and fluoroscopic studies confirm that combining KA with an MP insert improves the reproduction of physiological joint motion, closely approximating the biomechanics of a native knee [26, 45].

This study aims to evaluate the clinical efficacy of TKA performed with KA in combination with an MP‐TKA. The primary objective is to assess this technique's clinical and functional outcomes. Secondary objectives include analyzing the complication and short‐term revision rates and comparing clinical outcomes between patients with preserved versus sacrificed posterior cruciate ligament (PCL).

## MATERIALS AND METHODS

### Search strategy and study selection

**Figure 1:**
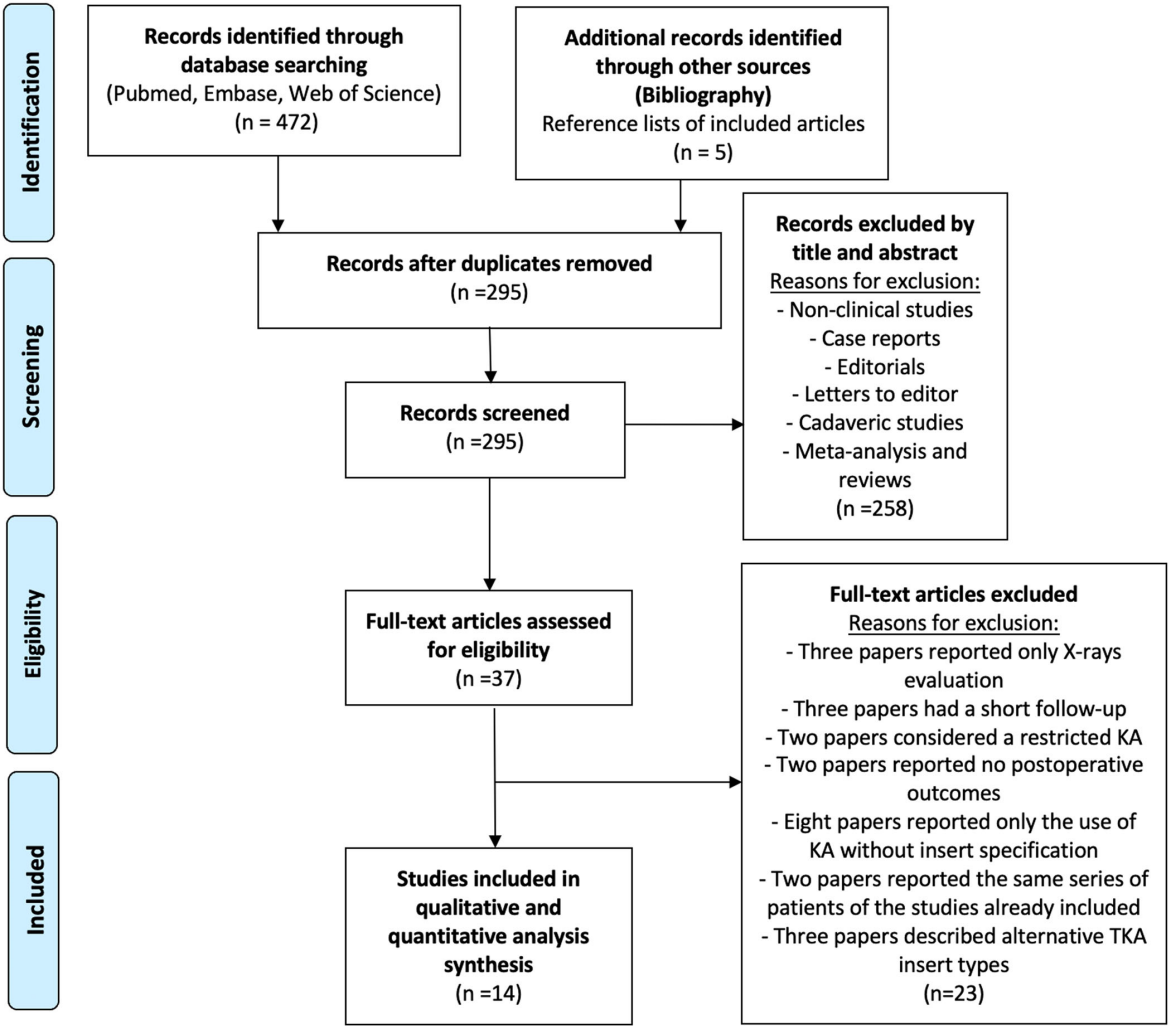
Preferred reporting items for systematic review and meta‐analyses (PRISMA) flow diagram of the studies included in the analysis. KA, kinematic alignment; TKA, total knee arthroplasty.

A comprehensive literature search was conducted across PubMed, Embase and Web of Science databases up to 31 January 2025, following the Preferred Reporting Items for Systematic Review and Meta‐Analysis (PRISMA) criteria [29] (Figure 1). The search utilized Boolean operators (‘OR’, ‘AND’) with terms such as ‘medial pivot’, ‘medial congruent’, ‘sphere’, ‘kinematic alignment’, ‘KA’, ‘total knee replacement’, ‘TKR’, ‘total knee arthroplasty’, and ‘TKA’. The reference lists of eligible studies were also manually screened to identify additional relevant articles. Two independent reviewers (G.C. and F.B.) performed title and abstract screening, followed by full‐text assessment. Discrepancies were resolved through discussion with a senior reviewer (L.S.). The protocol of this systematic review was registered in PROSPERO (CRD42022343517) [46].

### Inclusion and exclusion criteria

Studies were eligible if they included patients aged 40–90 years with end‐stage primary knee OA and a minimum follow‐up of 1 year. Studies involving secondary OA, such as post‐traumatic or inflammatory arthritis, were excluded. Only studies evaluating the clinical outcomes of primary, unrestricted KA using an MP insert were considered. Studies that applied restricted KA, MA or alternative insert designs such as cruciate‐retaining (CR), bicruciate‐retaining or posterior‐stabilized (PS) were excluded. Comparative analyses were preferred but not mandatory. The primary outcome was the clinical assessment using validated patient‐reported outcome measures (PROMs), including a ROM, Forgotten Joint Score (FJS), Knee Society Score (KSS), functional KSS (fKSS) and Oxford Knee Score (OKS). Secondary outcomes included complication rates, reoperation rates and subgroup comparisons of PCL preservation versus sacrifice. Non‐clinical studies, case reports, editorials, letters to the editor, cadaveric studies and non‐English publications were excluded. Studies with fewer than ten patients or a follow‐up period of less than one year were also excluded to ensure the robustness of the included evidence.

### Data extraction

Two independent reviewers (G.C. and F.B.) extracted data using a standardized spreadsheet. The extracted information included study characteristics (year of publication, first author, study design, TKA brand, comparative group), demographic details (number of patients, age, body mass index [BMI], gender distribution, follow‐up duration) and clinical outcomes (ROM, FJS, OKS, KSS and fKSS). Continuous variables were recorded as means with standard deviations (SDs), while categorical data were documented as frequencies and percentages. Any discrepancies were resolved by a senior author (L.S.).

### Quality evaluation

**Figure 2:**
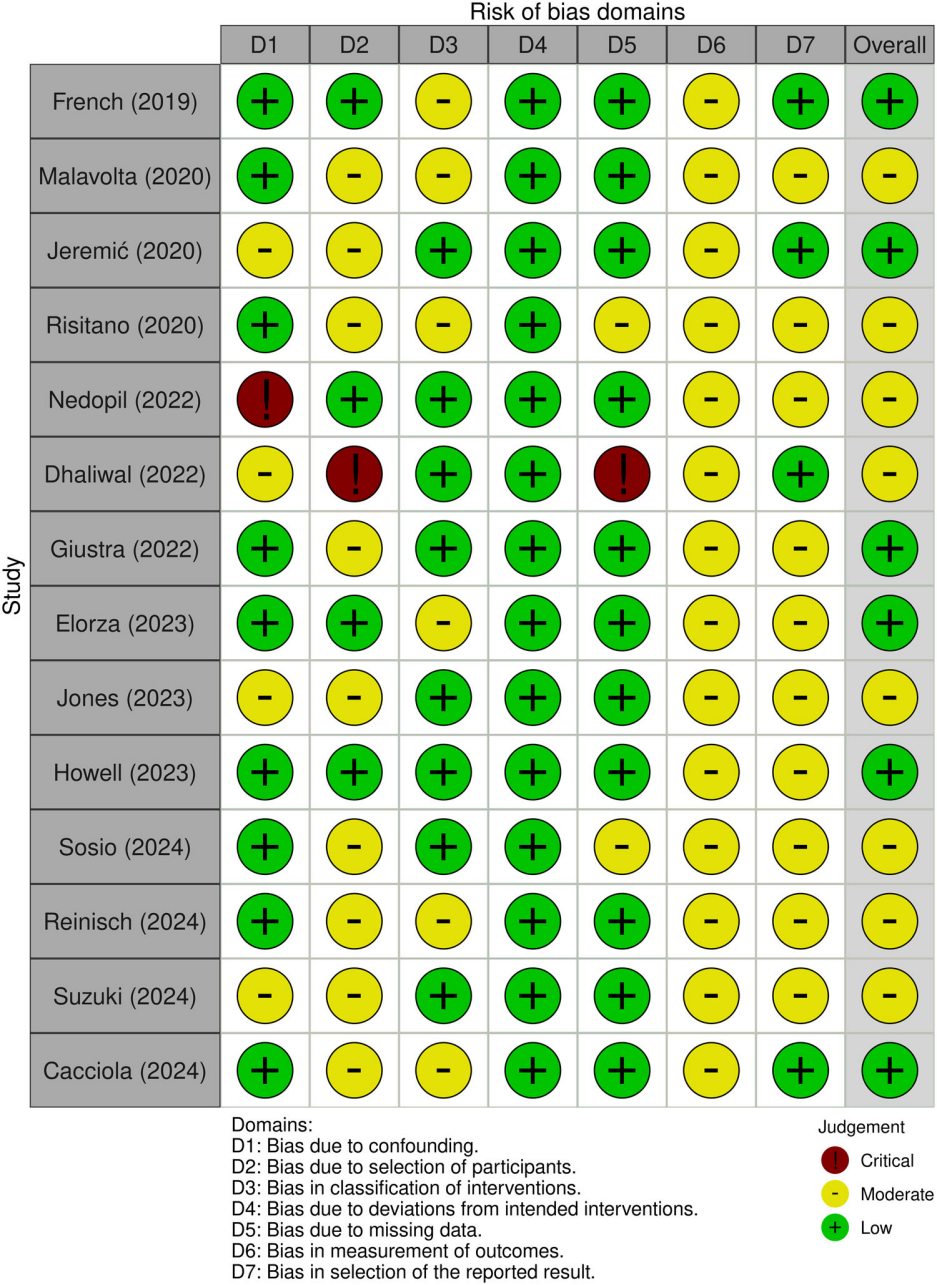
Assessment of the risk of bias of the individual studies included in the analysis according to the ROBINS‐I tool.

The methodological quality of the included studies was evaluated using the Oxford Centre for Evidence‐Based Medicine (2011) classification [5]. The Risk of Bias in Non‐Randomized Studies of Interventions (ROBINS‐I) tool was applied to assess potential bias (Figure 2) [49]. Two independent reviewers (G.C. and F.B.) conducted the quality assessment, with a third author (L.S.) resolving any discrepancies. All authors were actively involved in the study design, data analysis, manuscript preparation and final revisions.

### Statistical analysis

Descriptive statistics were applied to all collected data. Continuous variables were summarized as means with SDs, and categorical variables were expressed as frequencies and percentages. The Shapiro–Wilk test was used to assess data normality. Parametric methods were applied based on the assumption of normal distribution.

A meta‐analysis used a random‐effects model with inverse variance weighting to generate pooled estimates for continuous outcomes. The mean change from preoperative to postoperative scores was the primary summary measure. When only minimum and maximum values were available, SD was estimated using the formula (max − min)/4, under the assumption of an approximately normal distribution and an adequate sample size, as commonly applied in meta‐analytic methods. SDs for pre‐post changes were calculated with an assumed correlation coefficient of 0.7. Heterogeneity across studies was assessed using Cochran's *Q* test (considered significant at *p* < 0.10) and Higgins' *I*² statistic. *I*² values were interpreted as follows: 0%–24.9% = no heterogeneity; 25%–49.9% = low heterogeneity; 50%–74.9% = moderate heterogeneity and >75% = high heterogeneity.

A sensitivity analysis was performed by excluding outlier studies to assess the results' robustness further. Outliers were defined as studies whose 95% confidence interval (CI) was outside the 95% CI of the pooled effect. The meta‐analysis results were calculated when outliers were identified, excluding the identified outlier studies.

A significance threshold of *p* ≤ 0.05 was applied. All statistical analyses were performed using R software (version 4.2.1) with the meta‐R package [52].

## RESULTS

### Literature search and eligible studies

The systematic literature search identified 472 studies, of which 37 full‐text articles were screened for eligibility. After applying inclusion and exclusion criteria, 14 studies [6, 9, 11, 12, 16, 18, 24, 25, 33, 36, 40, 41, 48, 50] were included in the final analysis. Five studies were non‐comparative [9, 33, 41, 48, 50], while nine [6, 11, 12, 16, 18, 24, 25, 36, 40] were comparative. Three comparative studies [6, 16, 18] presented data separately for specific groups, requiring individual subgroup analysis. Giustra et al. [16] examined the outcomes of MP‐KA TKA with retained versus sacrificed PCL. Howell et al. [18] investigated the impact of prosthetic trochlear angle orientation relative to the quadriceps line of force. Cacciola et al. [6] analyzed postoperative outcomes based on native versus reduced posterior tibial slope. Elorza et al. [11] compared two insert designs (ball‐in‐socket vs. medial congruent); however, only the medial congruent group was included due to the short follow‐up of the other cohort. Nedopil et al. [36] assessed surgical outcomes based on surgeon experience. Still, only the cohort operated on by experienced surgeons was included due to the limited follow‐up of the inexperienced group.

### Study quality and demographic characteristics

The included studies were published between 2019 and 2024, marking the emergence of MP‐KA TKA within the last five years. Nine studies were Level III evidence, and five were Level IV, with no higher‐level studies available at the review time. A total of 976 patients (976 TKAs) were initially analyzed, but 21 cases (2.2%) were excluded due to loss of follow‐up, resulting in a final sample of 955 patients. The mean patient age at surgery was 70.2 years (66.2–73.9), with an average BMI of 29.4 kg/m² (26.4–32.9). Women comprised 57.8% of the total cohort. The mean follow‐up duration was 24 months (12–47 months). All the study details and demographic data are reported in Table 1.

**Table 1:** Demographic details of the included studies.

First author (YoP)	Level of evidence	Comparative group	No. patients (knees), no. (%)	Patients lost to FU, no. (%)	Mean age (years) (SD or range)	Female, no. (%)	BMI (kg/m^2^) (SD, range)	Follow‐up (months) (SD or range)
French (2019)	III	Cruciate retaining KA	53 (53)	7 (13.2%)	69.5 (6.9)	30 (65.2%)	32.9 (9.1)	13.1 (10.3–18.4)
Malavolta (2020)	III	No control group	60 (60)	1 (1.7%)	69 (8.3)	33 (55.9%)	27.1 (1.8)	36 (6.3)
Jeremić (2020)	III	MP MA	24 (24)	0 (0%)	70.7 (6.7)	13 (54.2%)	30.6 (4.5)	12
Risitano (2020)	IV	No control group	15 (15)	0 (0%)	73.5 (65–80)	8 (53.3%)	30.7 (21.5–36.3)	12
Nedopil (2022)	III	Experienced versus unexperienced surgeon	30 (30)	0 (0%)	69 (7.6)	16 (53.3%)	30 (6.2)	17 (5)
Dhaliwal (2022)	IV	No control group	43 (43)	0 (0%)	70 (8)	21 (48.8%)	32 (7)	23 (9)
Giustra (2022)	III	PCL retained versus sacrificed	64 (64)	0 (0%)	73.4 (8.2)	37 (57.8%)	26.4 (4.4)	28.9 (24–33.5)
Elorza (2023)	III	Ball‐in‐socket liner versus medial congruent liner	25 (25)	0 (0%)	68 (8)	11 (44%)	30.9 (5.4)	15 (7)
Jones (2023)	III	Versus PS and CS KA	101 (101)	13 (12.9%)	66.2	57 (56.4%)	31.3	24
Howell (2023)	III	PTA medial or lateral to the QLF	147 (147)	0 (0%)	//	79 (53.4%)	29 (18–43)	47 (8)
Sosio (2024)	IV	No control group	55 (55)	0 (0%)	71.5 (8.4)	32 (58.2%)	29.3 (4.6)	24
Reinisch (2024)	III	MP MA	71 (71)	0 (0%)	71 (10)	41 (58%)	27.2 (5.9)	12
Suzuki (2024)	IV	No control group	193 (193)	0 (0%)	68.3 (8.9)	98 (50.8%)	32.9 (6.2)	24
Cacciola (2024)	III	Native versus reduced slope	95 (95)	0 (0%)	73.9 (6.1)	56 (58.9%)	27.4 (3.4)	24

*Note*: All the numbers are abbreviated at one decimal.

Abbreviations: //, not reported; BMI, body mass index; CS, condylar stabilized; FU, follow‐up; KA, kinematic alignment; MA, mechanical alignment; MP, medial pivot; no., number; PCL, posterior cruciate ligament; PS, posterior stabilized; PTA, prosthetic trochlear angle; QLF, quadriceps line force; SD, standard deviation; YoP, year of publication.

### Meta‐analysis of clinical outcomes

**Figure 3:**
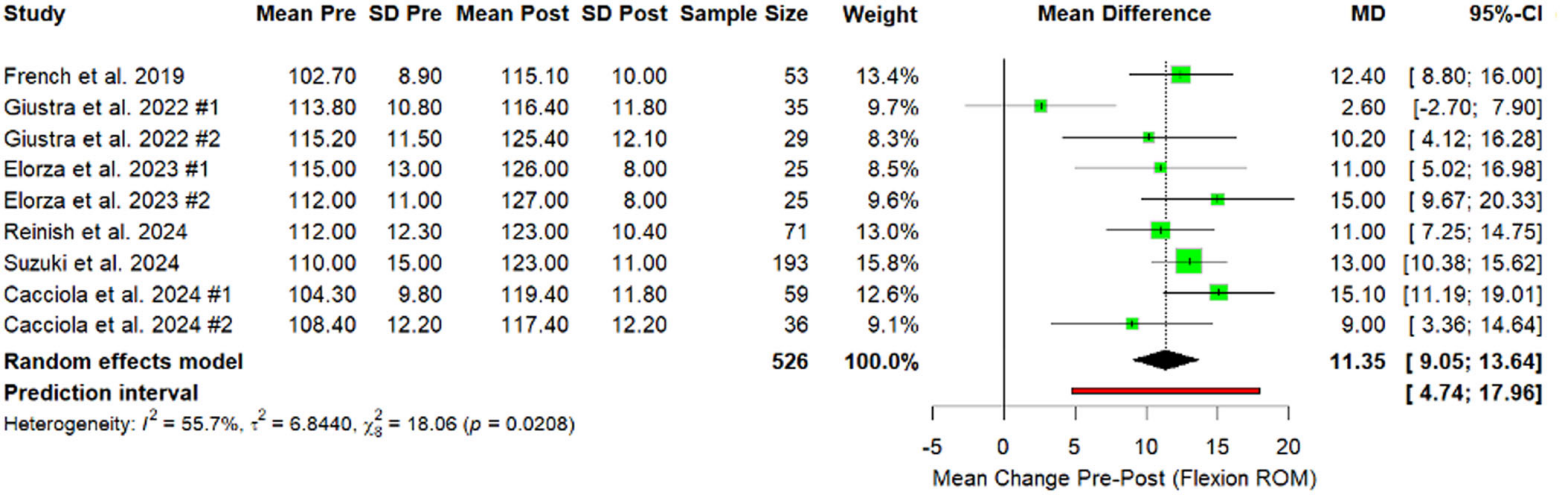
Forest plot. Comparison of Flexion Range results between preoperative and postoperative values. Giustra #1: PCL retained; Giustra #2: PCL sacrificed; Elorza #1: BiC; Elorza #2: MC; Cacciola #1: Reduced slope; Cacciola #2: Native slope. BiC, ball‐in‐socket; CI, confidence interval; MC, medial conformity; *p*, *p*‐value; PCL, posterior cruciate ligament; SD, standard deviation.

The meta‐analysis demonstrated statistically significant improvements in postoperative ROM and PROMs. The analysis revealed a moderate but statistically significant improvement in ROM, with a mean increase of 11.35° in flexion following MP‐KA TKA (Figure 3).

**Figure 4:**
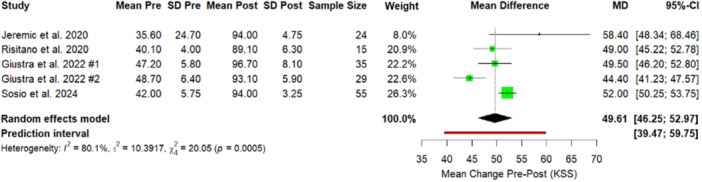
Forest plot. Comparison of KSS results between preoperative and postoperative values. Giustra #1: PCL retained; Giustra #2: PCL sacrificed. CI, confidence interval; KSS, Knee Society Score; *p*, *p*‐value; PCL, posterior cruciate ligament; SD, standard deviation.

**Figure 5:**
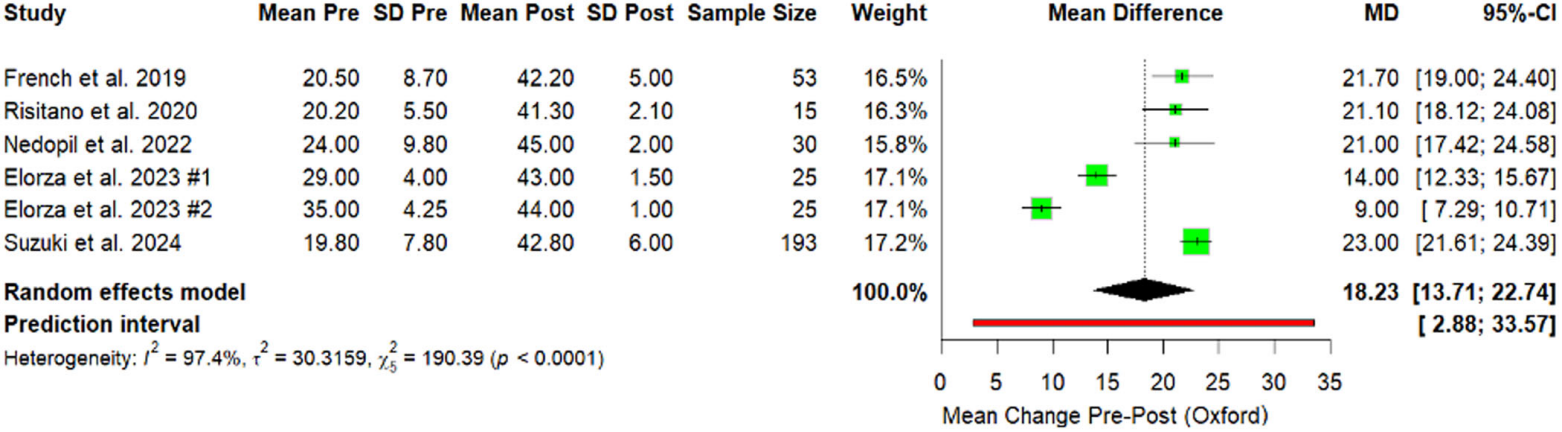
Forest plot. Comparison of Oxford results between preoperative and postoperative values. Elorza #1: BiC; Elorza #2: MC. BiC, ball‐in‐socket; CI, confidence interval; MC, medial conformity; *p*, *p*‐value; PCL, posterior cruciate ligament; SD, standard deviation.

**Figure 6:**
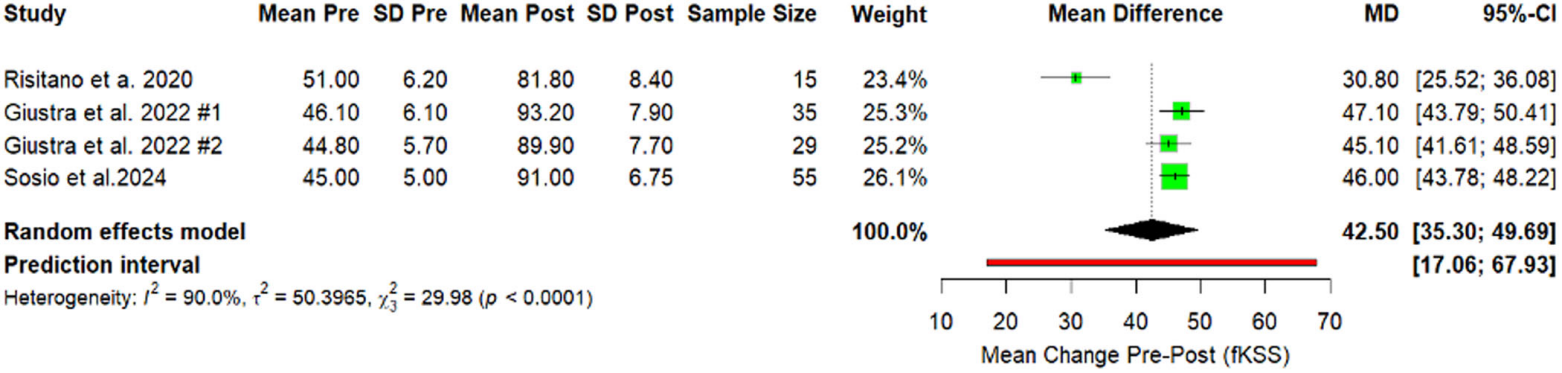
Forest plot. Comparison of functional KSS results between preoperative and postoperative values. Giustra #1: PCL retained; Giustra #2: PCL sacrificed. CI, confidence interval; KSS, Knee Society Score; *p*, *p*‐value; PCL, posterior cruciate ligament; SD, standard deviation.

Similarly, analysis of PROMs confirmed substantial functional recovery and all functional outcome measures demonstrated statistically significant improvements following the intervention. The KSS increased by 49.61 points (Figure 4), while the OKS showed a mean postoperative improvement of 18.23 (Figure 5). The fKSS also significantly increased, with a mean postoperative gain of 42.50 points (Figure 6). Additionally, the FJS reported a mean postoperative value of 75.6 (59.7–100), reflecting high levels of patient‐perceived joint integration and satisfaction. PROMs and ROM are reported in Table 2.

**Table 2:** PROMs and ROM.

Scores and ROM		French (2019)	Malavolta (2020)	Jeremić (2020)	Risitano (2020)	Nedopil (2022)	Dhaliwal (2022)	Giustra (2022) (PCL Retained)	Giustra (2022) (PCL Sacrificed)	Elorza (2023)	Jones (2023)	Howell (2023) (Medial to QLF)	Howell (2023) (Lateral to QLF)	Sosio (2024)	Reinisch (2024)	Suzuki (2024)	Cacciola (2024) (Native Slope)	Cacciola (2024) (Reduced Slope)	Numbers of patients	Average value (range)
Extension	Pre Mean (SD)	Na	Na	Na	Na	Na	8 (7)	Na	Na	8 (6)	5.08	11 (7)	13 (7)	Na	Na	8 (7)	Na	Na	496	8.5 (5.1–13)
	Post Mean (SD)	Na	0 (1.5)	Na	Na	Na	Na	Na	Na	0 (1)	−0.06	Na	Na	Na	Na	1 (3)	Na	Na	319	1.2 (−0.06–2)
Flexion	Pre Mean (SD)	102.7 (8.9)	Na	Na	Na	Na	113 (6)	113.8 (10.8)	115.2 (11.5)	112 (11)	116	114 (7)	116 (4)	Na	112 (12.3)	110 (15)	104.3 (9.8)	108.4 (12.2)	645	111.3 (102.7 to 117)
	Post Mean (SD)	115.1 (10)	124 (8.2)	Na	123 (5.3)	Na	Na	116.4 (11.8)	125.4 (12.1)	127 (8)	132	Na	Na	Na	123 (10.4)	123 (11)	119.4 (11.8)	117.4 (12.2)	677	123 (115.1–132)
FJS	Post Mean (SD)	79.9 (20.4)	Na	77 (58–96)	79.3 (3.3)	81 (56–100)	83 (50)	63.3 (19.8)	59.7 (18.1)	75 (66–88)	Na	83	100	89.6 (84.8–92.6)	Na	69.2 (26.6)	67.3 (8.9)	65.6 (9.1)	737	75.6 (59.7–100)
OKS	Pre Mean (SD)	20.5 (8.7)	Na	Na	20.2 (5.5)	24 (9.8)	21(8)	Na	Na	35 (25–42)	Na	21 (9)	23 (7)	Na	Na	19.8 (7.8)	Na	Na	456	21.5 (19.8–35)
	Post Mean (SD)	42.2 (5)	Na	Na	41.3 (2.1)	45 (39–47)	44 (11)	Na	Na	44 (42–46)	Na	47	46	Na	Na	42.8 (6)	Na	Na	558	43.8 (41–48)
KSS	Pre Mean (SD)	Na	Na	35.6 (24.7)	40.1 (4)	Na	Na	47.2 (5.8)	48.7 6.4)	50 (43–70)	Na	32 (11)	33 (17)	42 (29–52)	Na	Na	Na	Na	330	38.8 (32 – 50)
	Post Mean (SD)	Na	Na	94 (79–98)	89.1 (6.3)	Na	Na	96.7(8.1)	93.1 (5.9)	Na	Na	Na	Na	94 (85.5–98.5)	Na	Na	Na	Na	158	94 (89.1–96.7)
fKSS	Pre Mean (SD)	Na	Na	Na	51 (6.2)	Na	Na	46.1 (6.1)	44.8 (5.7)	Na	67.2	Na	Na	45 (40–60)	Na	Na	Na	Na	222	54.4 (44.8 – 67.2)
	Post Mean (SD)	Na	Na	Na	81.8 (8.4)	Na	Na	93.2 (7.9)	89.9 (7.7)	Na	85.8	Na	Na	91 (73–100)	Na	Na	Na	Na	222	88.5 (81.8–91)

*Note*: All numbers are abbreviated at one decimal.

Abbreviations: FJS, Forgotten Joint Score; fKSS, Functional Knee Society Score; KSS, Knee Society Score; Na, not available; OKS, Oxford Knee Score; PCL, posterior cruciate ligament; Post, postoperatively; Pre, preoperatively; PROM, patient‐reported outcome measure; QLF, quadriceps line force; ROM, range of motion; SD, standard deviation.

### Sensitivity analysis

**Figure 7:**
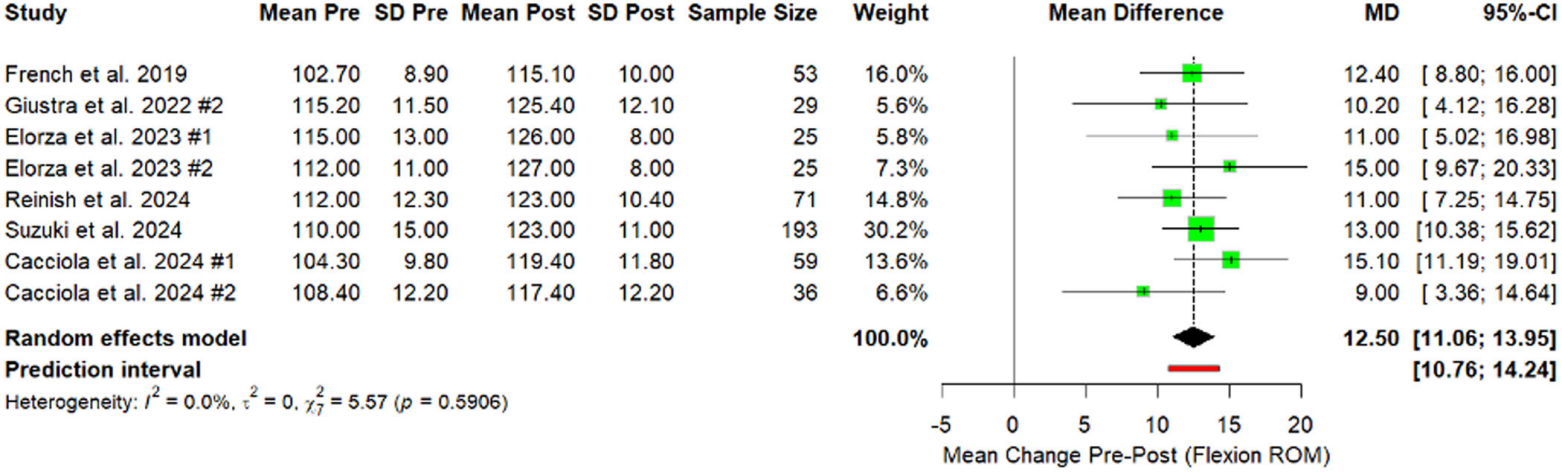
Forest plot. Sensitivity analysis. Comparison of flexion range results between preoperative and postoperative values after exclusion of outlier studies. Giustra #2: PCL sacrificed; Elorza #1: BiC; Elorza #2: MC; Cacciola #1: Reduced slope; Cacciola #2: Native slope. BiC, ball‐in‐socket; CI, confidence interval; MC, medial conformity; *p*, *p* value; PCL, posterior cruciate ligament; SD, standard deviation.

The sensitivity analysis was performed to evaluate the influence of outlier studies on the pooled effect estimates. No outliers were found for the outcomes of KSS and fKSS. One group in one study [16] was found to be an outlier for the flexion ROM outcome. Excluding this group reduced heterogeneity markedly from 55.7% to 0% and increased the mean difference (MD) from 11.35 (95% CI: 9.05–13.64) to 12.50 (95% CI: 11.06–13.95) (Figure 7). This finding suggests that variability in flexion outcomes was primarily driven by differences in PCL management strategies and surgical techniques reported in Giustra et al. [16] compared to the other included studies.

**Figure 8:**
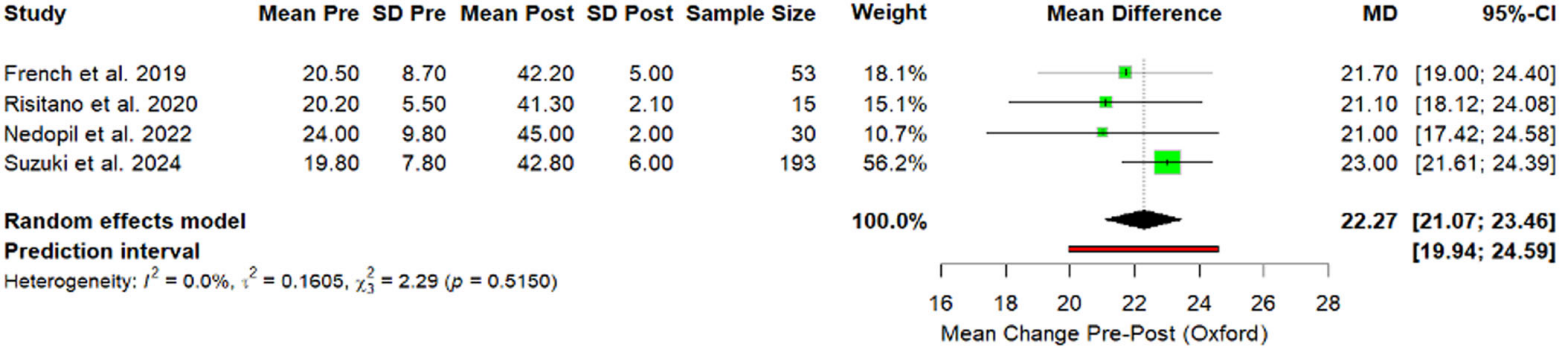
Forest plot. Sensitivity analysis. Comparison of Oxford results between preoperative and postoperative values after exclusion of outlier studies. CI, confidence interval; *p*, *p* value; SD, standard deviation.

Two outlier groups in the Elorza et al. [11] study were identified for the OKS. Their exclusion led to a complete elimination of heterogeneity (from 88.4% to 0%) and produced a statistically significant increase in the MD from 18.23 (95% CI: 13.7–22.7) to 22.27 (95% CI: 21.07–23.46) (*p* < 0.05) (Figure 8).

These sensitivity analyses indicate that excluding outliers enhances the homogeneity and precision of the pooled estimates and reveals a more pronounced postoperative improvement, thereby confirming the effectiveness of the MP design in improving patients' clinical outcomes after TKA.

### Complications

Postoperative complications were reported in 10 out of 14 studies [6, 9, 12, 16, 24, 25, 33, 36, 41, 48].

The estimated pooled incidence of complications was 1.6% (95% CI: 0.9%–2.4%).

The most commonly observed complication was postoperative stiffness, with seven reported cases; two of these required manipulation under anaesthesia [12], while five cases occurred in the study by Giustra et al. [16], distributed between PCL‐retained (three cases) and PCL‐sacrificed (two cases) groups.

Additionally, Malavolta et al. [33] described one case of a thromboembolic event requiring intensive care management. Importantly, no cases of aseptic or septic implant loosening, reoperations or revision surgeries were recorded during the follow‐up period.

### PCL retention versus sacrificed

Data on PCL status were reported in 13 studies (946 knees), while one study [36] did not specify PCL management. Suzuki et al. [50] noted that in their series, the PCL was sacrificed in 59% of cases based on intraoperative assessment; however, their data were excluded from subgroup analysis due to the lack of separate outcome reporting by PCL status. The remaining studies determined PCL retention or sacrifice based on surgeon preference and implant design characteristics. Implant characteristics and PCL status are summarized in Table 3.

**Table 3:** Type of TKA, implant characteristics and PCL retention or sacrifice.

First author (YoP)	Implant	No. patients (knees), no.	PCL ligament retained (yes, no)	Patellar resurfacing (yes, no)
French (2019)	Saiph (MathOrtho)	53 (53)	Yes	/
Malavolta (2020)	GMK Sphere (Medacta)	60 (60)	No	No
Jeremić (2020)	GMK Sphere (Medacta)	24 (24)	No	No
Risitano (2020)	Persona MC (Zimmer)	15 (15)	Yes	Yes
Nedopil (2022)	GMK Sphere (Medacta)	30 (30)	/	/
Dhaliwal (2022)	GMK Sphere (Medacta)	43 (43)	Yes	Yes
Giustra (2022)	Persona MC (Zimmer)	64 (64)	Retained in 35, sacrificed in 29	No
Elorza (2023)	GMK Sphere (Medacta)	25 (25)	Yes	Yes
Jones (2023)	GMK Sphere (Medacta)	101 (101)	No	/
Howell (2023)	Persona MC (Zimmer)	147 (147)	Yes	Yes
Sosio (2024)	GMK Sphere (Medacta)	55 (55)	No	No
Reinisch (2024)	GMK Sphere (Medacta)	71 (71)	No	/
Suzuki (2024)	GMK Sphere (Medacta)	193 (193)	59% sacrificed, 41% retained	90% resurfaced, 10% not resurfaced
Cacciola (2024)	Persona MC (Zimmer)	95 (95)	Yes	no

Abbreviations: /, no data reported; no., numbers; PCL, posterior cruciate ligament; TKA, total knee arthroplasty; YoP, year of publication.

Patients with sacrificed PCL demonstrated significantly greater postoperative maximum flexion (125.4° ± 12.1° vs. 116.4° ± 11.8°, *p* < 0.001). However, no significant differences were observed between groups for other outcome measures, including FJS (59.7 ± 18.1 vs. 63.3 ± 19.8, *p* = 0.401), KSS (93.1 ± 5.9 vs. 96.7 ± 8.1, *p* = 0.455) and fKSS (89.9 ± 7.7 vs. 93.2 ± 7.9, *p* = 0.351), suggesting that PCL management may not substantially impact subjective functional outcomes; however, caution is warranted, as the statistical power to detect subtle differences may be limited.

These results support the potential efficacy of MP‐KA TKA in restoring knee biomechanics, improving functional scores and maintaining favourable implant survival rates over the observed follow‐up period.

## DISCUSSION

This systematic review and meta‐analysis provide robust evidence supporting the clinical effectiveness of MP‐KA TKA. The findings confirm significant improvements in ROM, functional scores and patient‐reported outcomes. The results indicate a substantial gain in flexion and extension postoperatively, with PROMs demonstrating high levels of functional recovery. Additionally, the absence of aseptic loosening and reoperations supports the safety and durability of this approach in the short‐ to mid‐term follow‐up [6, 9, 11, 12, 16, 18, 24, 25, 33, 36, 40, 41, 48, 50].

The design evolution of TKA has focused on improving native knee kinematics and functional outcomes [13, 21, 28, 30, 42]. While MA‐TKA was historically the standard, its tendency to impose non‐physiological joint mechanics has led to increased interest in KA, which aims to restore the native joint line and ligament balance, enhancing proprioception and reducing soft tissue strain [13, 21, 28, 30, 38, 42].

Early KA‐TKA studies predominantly used CR inserts, assuming that preserving the PCL would maintain normal knee motion [10, 17, 35]. However, the transition to MP inserts was driven by their superior ability to replicate native biomechanics, ensuring medial compartment stability while permitting controlled lateral femoral rollback [1, 7, 17, 26, 44, 45]. Unlike traditional designs, MP implants prevent excessive translation and paradoxical femoral movement, as demonstrated in fluoroscopic and gait analysis studies [1, 3, 7, 17, 26, 44, 45]. The MP design constrains the medial femoral condyle while allowing physiological lateral rollback, improving proprioception and reducing instability risks [1, 7, 44]. Additionally, MP implants have been associated with lower polyethylene wear and reduced revision rates compared to CR and PS designs, reinforcing their role also in modern KA‐TKA, particularly in high‐demand patients [8, 17, 35, 43, 45].

The clinical success of KA‐TKA with MP implants has been supported by multiple studies demonstrating significant improvements in PROMs and functional assessments [6, 11, 25, 26, 50]. All analyzed functional scores in this investigation showed statistically significant postoperative gains, reinforcing the effectiveness of this approach.

The FJS, which assesses patients' perception of knee integration in daily activities, reported postoperative values comparable to those in previous systematic reviews despite the lack of preoperative data [41, 42, 44]. Similarly, the OKS and KSS showed substantial improvements, indicating better knee function, pain relief and overall patient satisfaction [12, 26, 30, 41]. High OKS scores reflect enhanced daily function, including stair climbing and prolonged ambulation.

Gait analysis studies further confirm that KA‐TKA with MP implants promotes a more natural walking pattern, reduces quadriceps co‐contraction and improves stance‐phase stability compared to MA‐TKA [2, 18, 26, 45, 47]. These biomechanical benefits may lower the risk of compensatory movement disorders, contralateral joint overload and lower back pain [2, 47].

Longitudinal follow‐up data indicate that KA‐TKA sustains high patient satisfaction, a complication rate and implant survival, like MA‐TKA [13, 28, 30, 38]. Additionally, reduced anterior knee pain and patellofemoral complications may be attributed to improved trochlear groove conformity and controlled patellar tracking [2, 18, 31, 47]. These findings highlight the potential superiority of KA‐TKA with MP prostheses in restoring knee biomechanics and optimizing patient outcomes.

The long‐term durability of KA‐TKA remains a critical consideration, particularly regarding aseptic loosening and implant stability [13, 14, 15, 20, 21, 22, 28, 30]. The absence of loosening or implant failure at short and mid‐to‐long‐term follow‐up is reassuring, suggesting stability comparable to MA‐TKA [15, 20, 22]. Clinical data have not substantiated concerns about altered ligamentous tension and joint line positioning in KA‐TKA [13, 15, 20, 21, 22, 28]. The medial‐conforming nature of MP implants may enhance tibial fixation by optimizing load distribution and minimizing shear forces [1, 3, 26, 45]. Long‐term studies confirm that the aseptic loosening rate of KA‐TKA is like MA‐TKA [20, 22]. Tibial component survivorship appears to depend on coronal alignment, implant selection and bone quality rather than alignment strategy alone [16, 20, 21, 22]. Additionally, the MP design may reduce polyethylene wear and the risk of secondary osteolysis compared to PS or CR designs by maintaining a stable medial contact point and allowing controlled lateral femoral rollback [1, 7, 8, 43, 44].

Overall, evidence supports KA‐TKA with MP implants as a biomechanically stable and functionally reliable option, with aseptic loosening rates comparable to MA‐TKA [12, 20, 24, 33, 36, 41].

The debate on PCL retention in KA‐TKA remains unresolved, as it influences joint mechanics and implant performance [16, 19, 37]. PCL retention preserves the native tibial slope, optimizing sagittal plane biomechanics and potentially improving posterior stability [19, 27, 39]. However, not all implants are designed for this approach. The Medacta Sphere insert, initially intended for PCL sacrifice, was later adapted with a ball‐in‐socket configuration to allow retention while minimizing impingement risks [19, 33, 45, 50].

Our findings suggest that PCL sacrifice may provide slightly improved postoperative flexion due to a more predictable rollback mechanism and eliminating variability in PCL tensioning. However, long‐term data cannot determine definitive functional advantages [4, 16, 37].

Despite the strengths of this study, several limitations must be considered.

First, the included studies predominantly comprise Level III or IV evidence, limiting the strength of the conclusions and their generalizability to broader patient populations.

Furthermore, the relatively short follow‐up period of the included studies (minimum one year) limits the ability to draw conclusions about long‐term implant survivorship and durability. Additionally, variability in implant designs, surgical techniques and patient selection criteria across studies introduces potential confounding factors that may influence the reported outcomes. Differences in component positioning, soft tissue balancing strategies and rehabilitation protocols further complicate direct comparisons between studies. Moreover, the lack of standardized radiographic and kinematic assessments limits the ability to perform a detailed analysis of implant stability and wear characteristics. Finally, the absence of a direct comparison group using alternative alignment strategies or implant designs precludes definitive conclusions regarding the relative superiority of MP‐KA TKA. Additionally, the absence of reported aseptic or septic loosening and reoperations across all included studies may reflect an underreporting bias or publication bias, and therefore, the complication rates should be interpreted with caution. Future research should prioritize well‐designed, long‐term, randomized controlled trials with standardized methodologies to validate the clinical and biomechanical advantages of MP‐KA TKA. Large‐scale registry data and multicenter studies could provide further insights into implant survivorship, patient satisfaction and potential risk factors for failure.

## CONCLUSIONS

MP‐KA TKA represents a promising approach for optimizing knee biomechanics and enhancing patient outcomes. Combining unrestricted KA with an MP insert has significantly improved the ROM, functional scores and patient satisfaction. The low complication rate and absence of early implant failures further support the viability of this technique. However, ongoing research is needed to confirm its long‐term durability and to refine implant selection criteria for different patient phenotypes. Future comparative studies should aim to establish clear guidelines for PCL retention and further investigate the impact of KA on implant longevity.

## AUTHOR CONTRIBUTIONS

Francesco Bosco and Giorgio Cacciola have contributed substantially to conception and design, data acquisition, analysis and interpretation. They agree to be accountable for all aspects of the work and ensure that questions related to the accuracy or integrity of any part of the work are appropriately investigated and resolved. Virginia Masoni, Carmelo Burgio, Mariazzurra Carlino, Michele Centola, Ferdinando Tosto and Daniele Vezza have contributed substantially to the data analysis, interpretation and manuscript drafting. Lawrence Camarda and Luigi Sabatini have significantly contributed to revising the manuscript critically for important intellectual content, giving final approval for the publication of the version.

## CONFLICT OF INTEREST STATEMENT

The authors declare no conflicts of interest.

## ETHICS STATEMENT

Not applicable.

## Data Availability

The data set analyzed in this study is available from the corresponding author upon reasonable request.
